# Evaluation of Fiber Reinforced Cement Using Digital Image Correlation

**DOI:** 10.1371/journal.pone.0128644

**Published:** 2015-06-03

**Authors:** Garrett W. Melenka, Jason P. Carey

**Affiliations:** University of Alberta, Edmonton, Alberta, Canada; Northwestern Polytechnical University, CHINA

## Abstract

The effect of short fiber reinforcements on the mechanical properties of cement has been examined using a splitting tensile – digital image correlation (DIC) measurement method. Three short fiber reinforcement materials have been used in this study: fiberglass, nylon, and polypropylene. The method outlined provides a simple experimental setup that can be used to evaluate the ultimate tensile strength of brittle materials as well as measure the full field strain across the surface of the splitting tensile test cylindrical specimen. Since the DIC measurement technique is a contact free measurement this method can be used to assess sample failure.

## Introduction

The use of fiber reinforcements in cement based materials is increasing due to the ability to tailor cement and concrete characteristics to specific applications and environments [1]. The addition of short fibers to cement-based products improved ductility, fracture toughness and durability [2–6]. Short fibers are a dry product and therefore can more readily be blended with dry cement. Furthermore, short fibers can also be selected to be chemically inert; therefore, their addition will not have an impact on vital cement chemical properties. Finally, short fibers can also be selected for high temperature environments [2, 3]; for instance, glass fibers have a melting point of approximately 860°C and carbon fibers melt at 3500°C. Therefore, short fiber reinforcements can be utilized in harsh environments where cement based products are commonly used while still improving cement mechanical properties.

A splitting tensile test, also known as the Brazilian test method, is a common approach for evaluating the tensile strength of brittle materials [7–11], but can also be used to evaluate the elastic constants and deformation of brittle materials [8, 9, 12]. The splitting test configuration consists of a cylindrical test sample that is loaded with diametrical compression. The applied compressive load induces tensile stresses perpendicular to the applied load. Tensile failure occurs since brittle materials have a tensile strength that is much less than compressive strength. This indirect method has been developed due to challenges in directly evaluating the mechanical properties of low tensile strength brittle materials [8–10]. In addition, an analytical stress solution exists for cylindrical samples under the loading conditions used in the splitting tensile test [7, 9–11]. The analytical stress solution has been compared and validated with experiments [7, 9].

Conventional splitting tensile tests utilize strain gauges at discrete locations to evaluate sample mechanical properties; however, the entire sample surface strain field cannot be evaluated with this standard measurement approach. Contact free measurement methods, such as a holographic interferometry, have been used to evaluate cement based materials [12]. This method only allows for strain to be measured up to the point of initial crack propagation. Since holographic interferometry is highly sensitive to rigid body motions, crack propagation cannot be investigated as the samples are not stable after failure occurred[13].

Digital image correlation (DIC) is an optical measurement technique that offers increased capabilities to conventional measurement techniques [13, 14]. DIC is a full-field strain measurement method that uses a random speckle pattern applied to the surface of an object to measure deformation and strain. This technique has been used for a variety of applications [13, 14]. Deformation is measured by using optical cross-correlation algorithms to track contrast features on the specimen surface between subsequent images. Cement samples have been measured using DIC [15]. The DIC measurement method has been used with brittle epoxies using a splitting tensile test in order to determine sample elastic properties [9]. One advantage of the DIC method is that it is less sensitive to bulk motion than interferomic full field strain measurement methods [13]. As a result, the DIC measurement method can be used to measure sample failure.

The goal of this study is to evaluate the effect of short fibers on the mechanical behavior of cement using an indirect splitting tensile test in conjunction with the DIC optical measurement method to evaluate sample displacement and strain as well as crack propagation and failure process. A custom splitting tensile- DIC test apparatus was designed and built to carry out cement sample mechanical tests. A splitting tensile test was performed on the cement samples in order to determine the load-strain behavior of the cement samples. An ASTM standard that uses the splitting tensile test method exists for the evaluation of the ultimate tensile strength of cement samples [ASTM C496]; however, no standard exists for evaluating the mechanical properties of a cement sample using the splitting tensile method.

## Methods

The deformation and strain of cylindrical cement samples were evaluated using a splitting tensile-digital image correlation test method. The digital image correlation measurement method was selected for evaluating the cement samples since this technique provides full field strain measurement of the test samples. In addition, the sample preparation and setup method is simple to perform compared to conventional cement tensile testing preparation methods since the instrumentation of devices like strain gauges is not required.

### Test Apparatus

**Fig 1:**
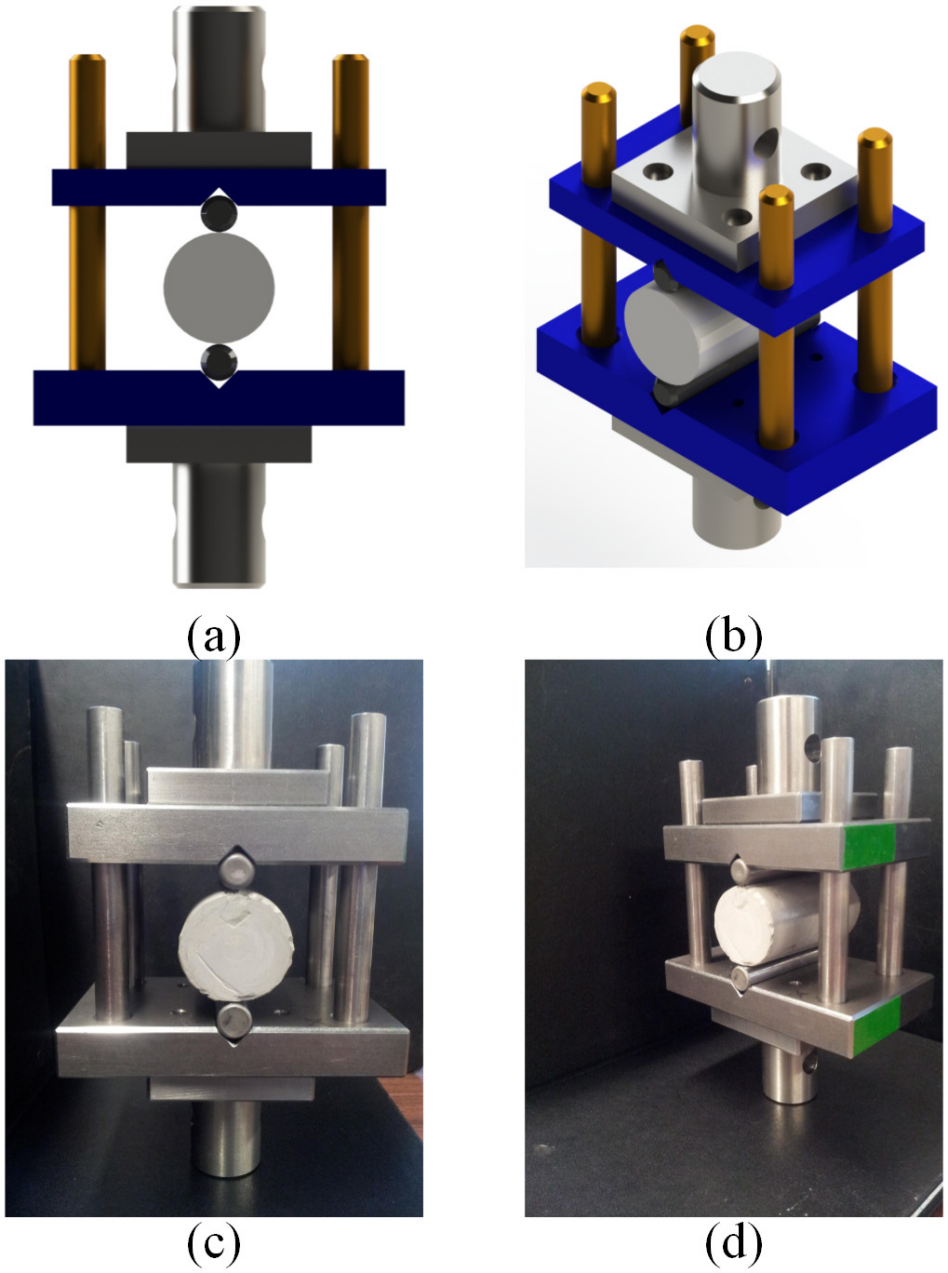
Cement sample splitting tensile test apparatus (a) front view rendering (b) isometric view rendering (c) front view of test apparatus (d) isometric view of test apparatus.

The custom test apparatus for performing the splitting tensile tests is shown in Fig 1. This figure shows front and isometric views of the test apparatus for both the design and the final machined apparatus. A similar splitting tensile test apparatus was utilized by Jianhong *et al*. for the estimation of the tensile elastic moduli of materials such as marble, limestone, sandstone and granite [8]. The splitting tensile apparatus uses two high strength 12.7mm x 76.2mm steel dowels to provide loading to the test sample. The high strength dowels provide concentrated loading to the test samples. This loading configuration was selected to aid in the measurement of the strain distribution across the surface of the test samples [8]. The test apparatus was designed for testing samples with a diameter of 38.1mm (1.5”) and a length of 76.2mm (3”). The dimensions for cylindrical test specimen are specified by ASTM C192/C192M Standard Practice for Making and Curing Test Specimen in the Laboratory.

### Cement Sample Preparation

**Fig 2:**
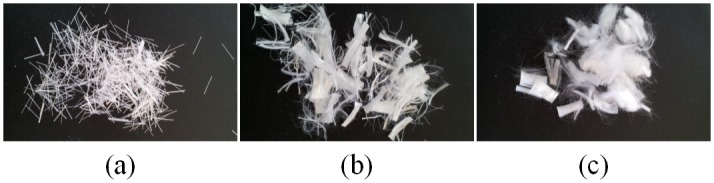
Short fiber additives (a) Nycon AR-DM (Fiberglass), (b) Nycon RC (Nylon), (c) Procon M (polypropylene).

Short fibers were added to cement in order to examine the effect of short fiber material and quantity on the mechanical properties of the fiber reinforced cement. For this study, three short fiber materials were selected, fiberglass, nylon and polypropylene (Nycon AR-DM, NyconRC Procon M, Nycon Corporation, Fair Hills, PA), since they will not chemically react with cement and are easily blended with cement Images of the three short fiber additives are shown in Fig 2. Additionally, the dimensions and mechanical properties of the short fiber additives are listed in Table 1. Oil Well G cement was used since it is commonly used in the oil and gas industry.

**Table 1:** Short fiber additives dimensions and mechanical properties.

Product Name	Material	Length (mm)	Diameter (μm)	Tensile Strength (MPa)	Elastic Modulus (GPa)	Specific Gravity
Nycon RC 3/4"	Nylon	19	9	300	2–3.6	1.15
Procon M	Polypropylene	19	38	170	4	0.91
Nycon-AR-DM	FiberGlass	13	10	2000	72	2.7

All cement samples were cured in molds made from ABS tubing. The ABS tubing had an inner diameter of 1.5” (38.1mm) and each mold was cut to allow for 3” (76.2mm) long samples to be manufactured. The dry cement, water and fibers were blended using a drink mixer (Hamilton Beach Drink Master Drink Mixer). All samples were mixed for two minutes using the drink mixer. Once the samples were mixed, they were poured into the ABS molds and allowed to cure for 72 hours. The quantities of cement, water and fibers used in this study are shown in Table 2. The mass fraction equation shown in Eq 1 was used to calculate the required mass of fibers. Five samples were manufactured for each cement and short fiber reinforcement combination.

**Table 2:** Cement and fiber reinforcement quantities.

Fiber	Volume Fraction %	Mass Fraction %	Oil Well G Mass (g)	Water Mass (g)	Mass Fibers (g)	Slurry Density (kg/m^3^)
Oil Well G	0	0.00	332.23	146.18	0	1914
Nycon RC	1	0.36	330.3	145.75	1.19	1909
Nycon AR-DM	0.5	0.42	330.9	146.08	1.39	1913
	1	0.85	329.51	145.98	2.8	1913
	2	1.69	326.94	145.79	5.53	1913
Procon M	1	0.29	330.38	145.7	0.96	1908
	5	1.47	323.08	143.82	4.75	1887

The mass fraction of the short fibers was calculated using Eq 1.

$$W_{f}=\frac{\frac{\rho_{f}}{\rho_{m}}}{\frac{\rho_{f}}{\rho_{m}}V_{f}+V_{m}}V_{f}$$ 1

Here, *ρ*_*f*_ is the fiber density, *ρ*_*m*_ is the cement density, *V*_*f*_ is the fiber volume fraction, and *V*_*m*_ is the cement volume fraction. The volume fractions shown in Table 2 were selected as these quantities of fibers could be blended with the cement. Higher volume fractions would not disperse uniformly within the cement.

### Mechanical Testing Procedure

#### Speckle Pattern Application

**Fig 3:**
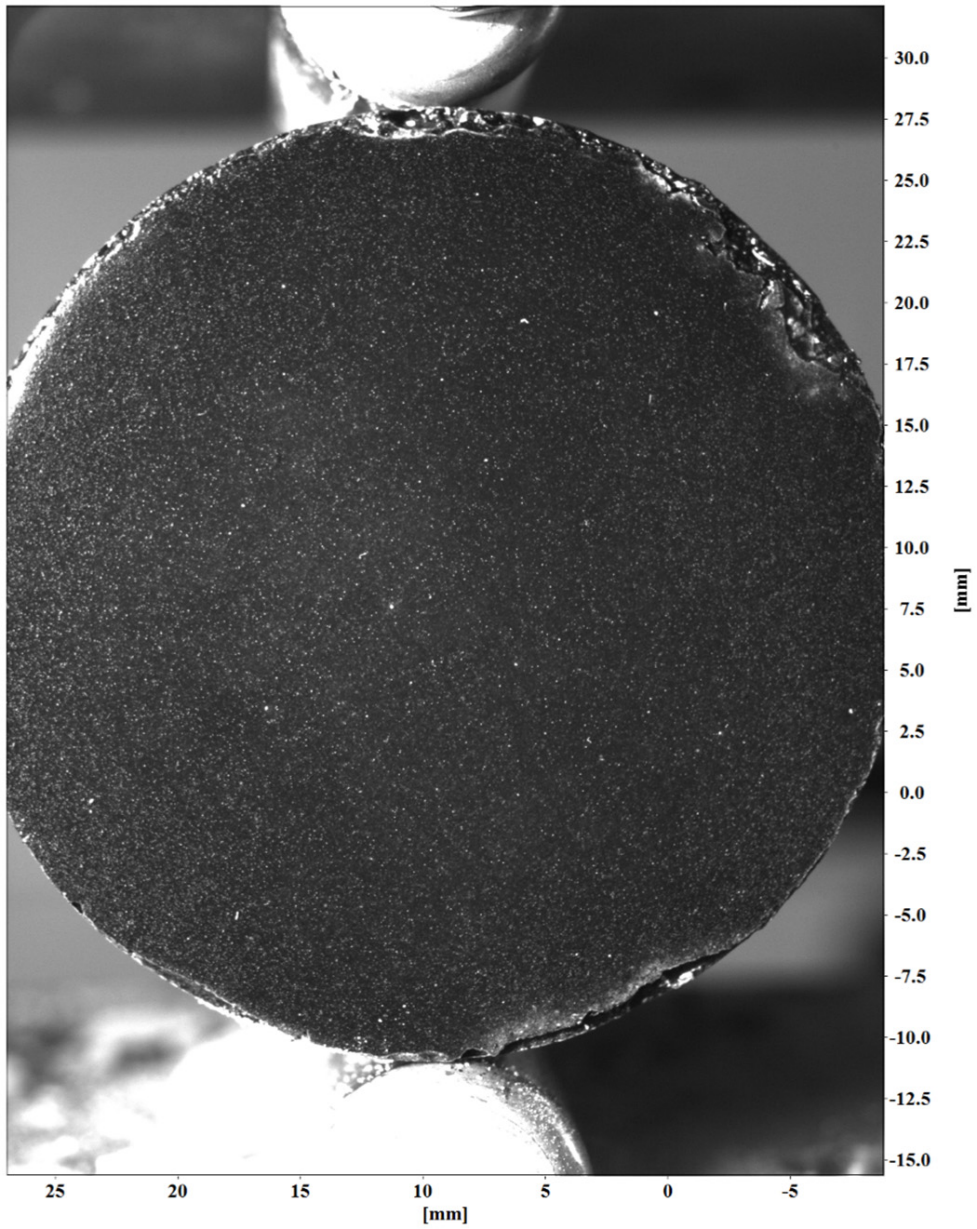
Cement test sample with speckle pattern applied to surface.

The cement samples were prepared for testing after curing and de-molding. A similar DIC sample preparation has been utilized by Leung and Melenka [16, 17]. The flat surface of each cement sample was first spray painted black using a flat black spray paint (Krylon 51602 Flat Black Interior- Exterior Paint) to increase contrast for the DIC optical measurements. After applying spray paint to the sample surface, an airbrush (Custom Micron-B, Iwata-Medea Inc., Portland, OR) was used to apply particles (Sphericel 110P8 25μm hollow microspheres) to the samples surface. The particles were mixed with a transparent base (W200 Transparent Base, Wicked Colours, East Granby CT) to ensure adhesion to the sample surface. A final prepared sample with particles is shown in Fig 3. This figure shows the contrast between the hollow microspheres and the surface of the cement sample.

**Fig 4:**
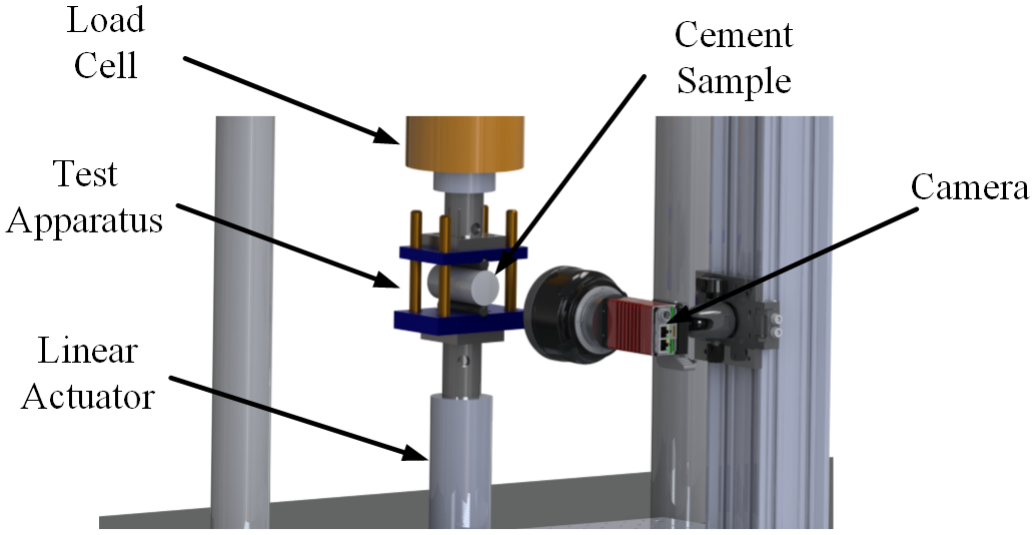
Cement sample splitting tensile test setup. This image shows the cylindrical test specimen located within the splitting tensile test apparatus. A compressive load is applied by the linear actuator and loads applied to the sample are measured with a load cell. A high resolution camera is used to measure the displacement and strain that occurs to the flat face of the cylindrical test sample.

The splitting tensile test setup is shown in Fig 4. This image shows the cement test sample within the splitting tensile test apparatus. The loads applied to the test specimen were measured using a load cell (661.12B, MTS, Minneapolis, MN, USA). Load cell data was recorded at a rate of 100 Hz using a National Instruments data acquisition system (NI-USB 6211 DAQ, National Instruments, Austin, TX). All test samples were imaged using a high resolution camera (AVT GX3300, Allied Vision Technology, Stadtroda, Germany). A 35mm camera lens (Nikon Nikkor AF-S DX 35mm f/1.8G, Nikon, Melville, NY) was used to collect images of the test samples. An f-stop number of 8 was used for all collected images to ensure appropriate contrast in the sample image. In addition to a 35mm lens, a 10mm extension tube was attached to the camera lens. The extension tube allowed for the cement sample to maximize the camera field of view as shown in Fig 3. Maximizing the cement sample within the camera field of view ensured that small deformations and strains could be detected using the DIC measurement technique.

Prior to starting the test, a preload was applied to all samples to ensure the test sample was secured within the testing apparatus. An initial loading rate of 1.2 mm / min was used following existing procedures [18] for metal fiber reinforced cement; however, this loading rate resulted in rapid failure of the cement test samples. As well, ASTM C496 prescribes a loading rate of 0.7 to 1.4 MPa / min; however this loading rate is used for determining the ultimate tensile strength of cements. Since the loading rate of 1.2mm/min resulted in rapid sample failure a loading rate of 0.12mm / min was used for the cement sample tests. This loading rate was chosen to allow for a large number of images to be collected during testing.

### Digital Image Correlation

**Fig 5:**
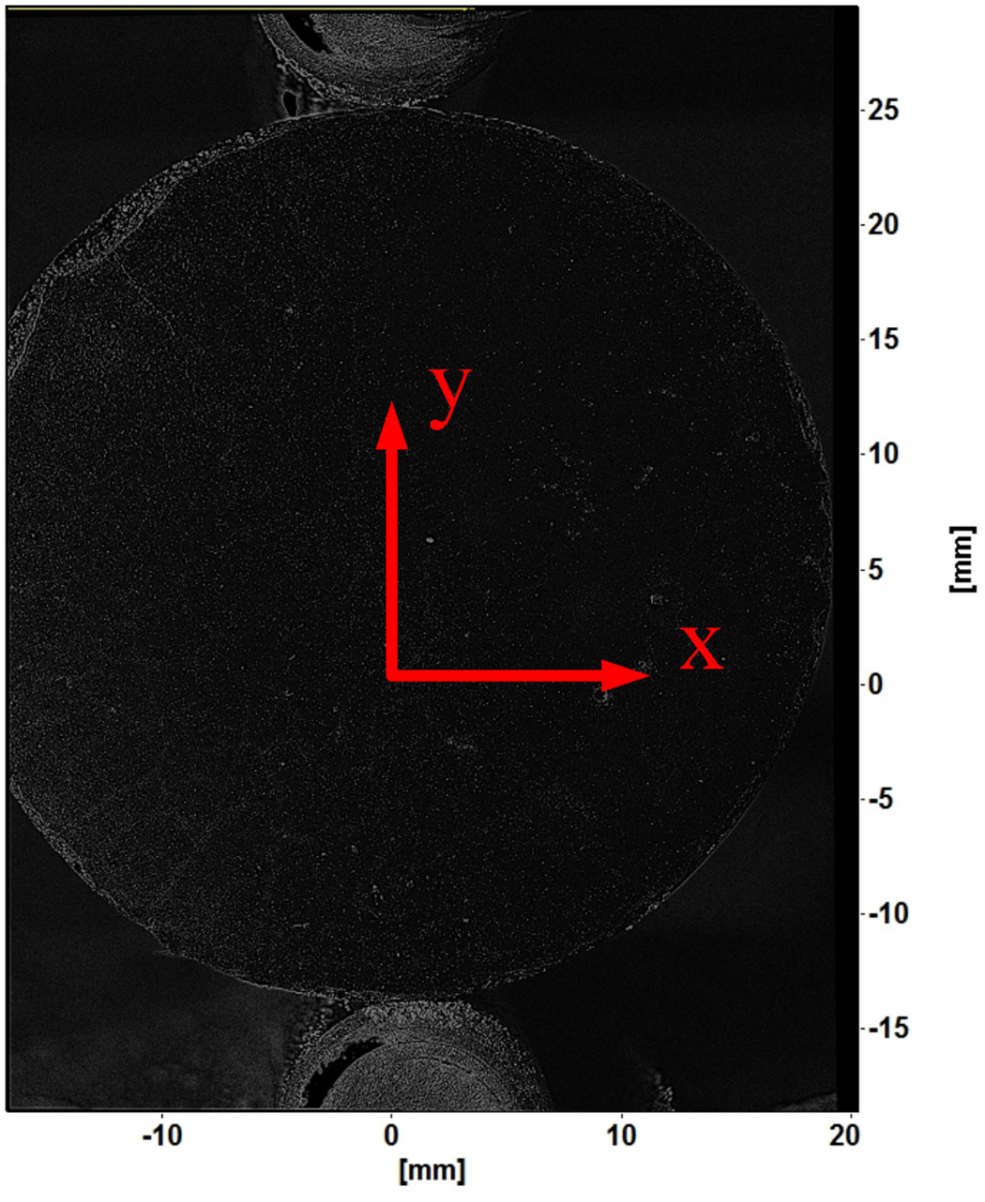
Example scaled cement sample image with coordinate system. Image contrast has been adjusted by applying sliding average and normalization filters.

The image sequences collected during the mechanical testing were processed using a commercial software package (LaVision GmbH DaVis 8.08. Gottingen, Germany, 2013). This software package was used to determine the deformation and strain for each test sample. Before the sample deformation can be calculated, all images are calibrated to correct distortions inherent in all camera lenses and scale the images into physical units (i.e. millimeters). An example of a scaled cement sample image is shown in Fig 5. This image also shows the defined coordinate system for each cement sample. The tensile load was applied to each sample along the *y*-axis. Tensile strain will occur along the *x*-axis.

**Fig 6:**
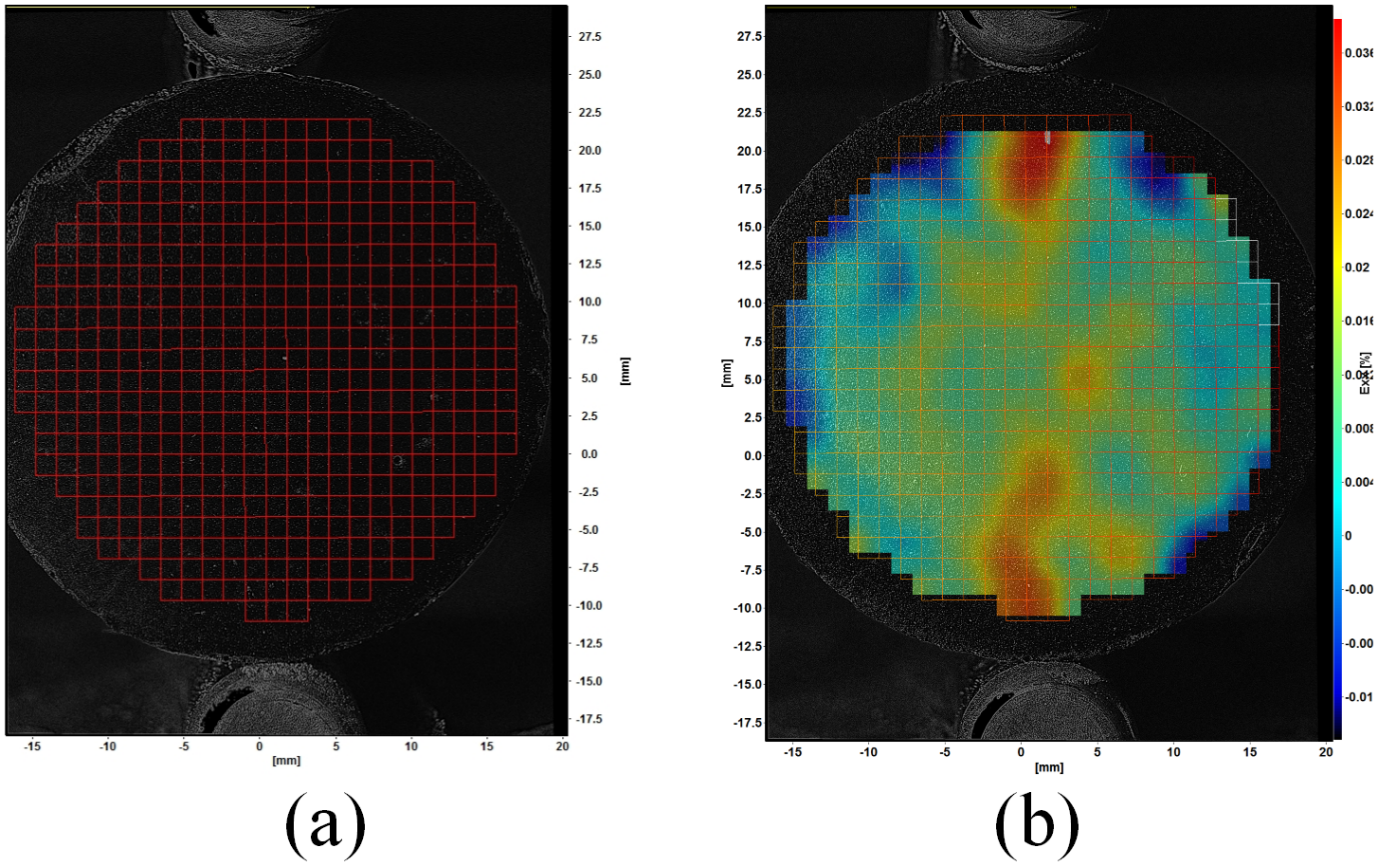
Example cement sample (a) with applied 128x128 pixel vector grid (b) with example strain field *ε*_*xx*_.

Image subsets, or window size, used to determine the 2D displacement of the cement samples were 128x128 pixels. The 128x128 interrogation windows were used to reduce errors in strain values caused by using smaller interrogation windows; this size has a 0.005 pixel 2D vector precision [19]. An example of the interrogation window grid used for strain measurement of the cement samples is shown in Fig 6(a). A circular geometric mask was applied to each sample image to ensure only the cylindrical cement sample was analyzed. A sliding average and max-min normalization filters were used to improve the contrast of the sample images. Fig 3 and Fig 5 show the effect of applying image contrast filters to the cement sample images.

Once all images were calibrated and preprocessed, the digital image correlation algorithm was used to determine the deformation and strain for each test sample. An example cement sample with strain pattern determined using the DIC measurement algorithm is shown in Fig 6(b). Fig 6(b) shows that the greatest strain occurs along the loading access of the sample (*y*-axis).

**Fig 7:**
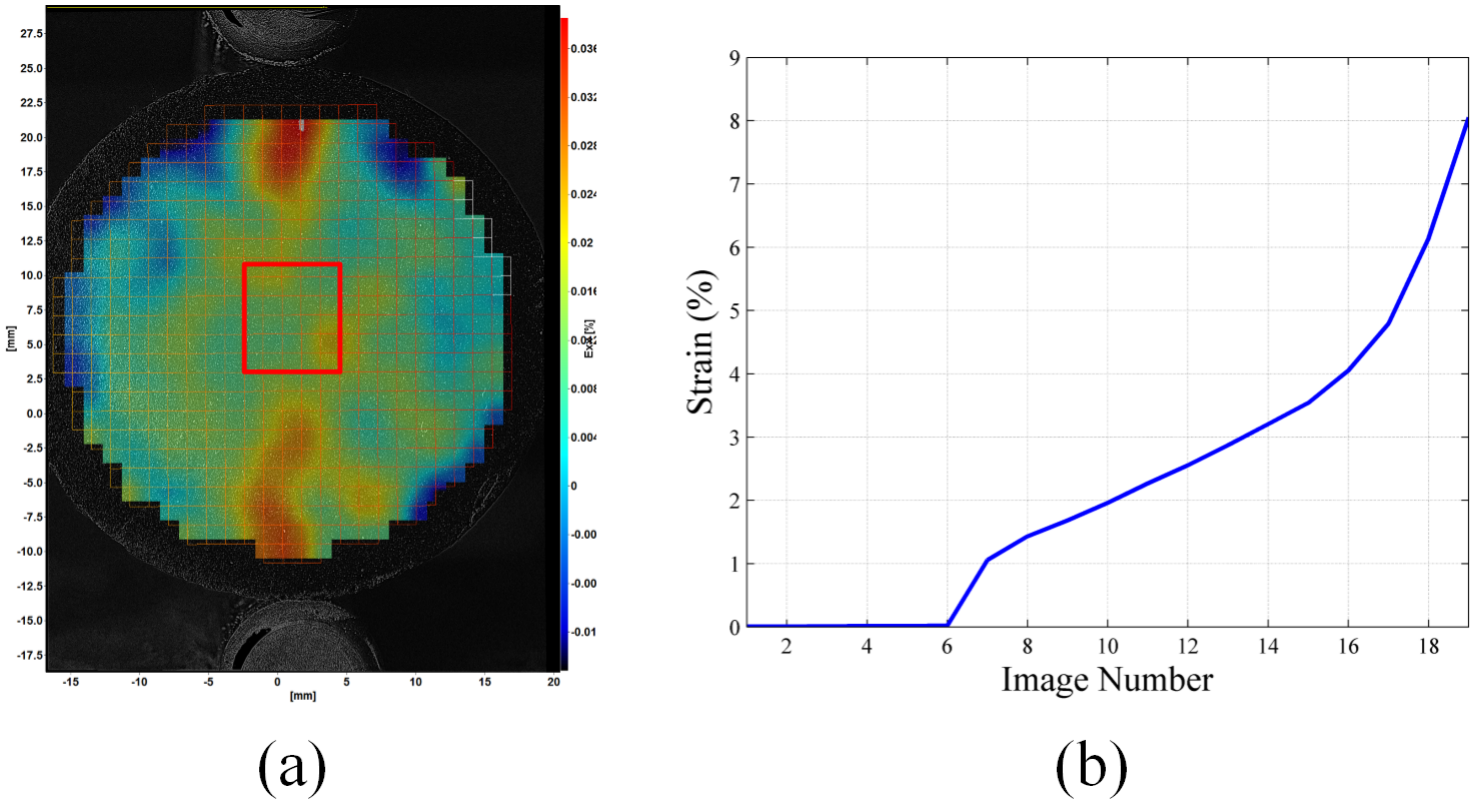
Cement sample strain quantification (a) example cement sample with rectangular region used to calculate average strain (b) plot of average strain- ε_*xx*_ vs cement sample image number.

To quantify the measured strain of each cement sample, a rectangular region at the center of the cement cylinder was selected. This is the location where maximum stress and strain will occur due to the applied compressive load [7, 9, 11]. An example of rectangular region used to calculate the average strain is shown in Fig 7(a). The resultant average tensile strain (*ε*_*xx*_) versus image number for this sample is shown in Fig 7(b). Fig 7(b) shows sample strain before and after failure. A significant increase in sample strain post-failure is shown in Fig 7(b) due to the presence of short reinforcing fibers.

To evaluate the load bearing capability of each of the fiber reinforced cement samples, toughness was calculated. Toughness was estimated by calculating the area under the load vs. strain curve for 2% strain for all samples. A similar approach for evaluating the toughness of fiber reinforced cement for three-point bend tests was performed by Felekoğlu *et al* [20].

Additionally, the splitting tensile stress for each sample was calculated using Eq 2 (ASTM C496), where *T* is the splitting tensile strength (MPa), *P* is the measured load applied to the sample (N), *l* is the length of the sample (mm), and *d* is the sample diameter (mm). The splitting tensile strength was used to compare the effect of short fiber reinforcement.

$$T=\frac{2P}{\pi ld}$$ 2

## Results

**Fig 8:**
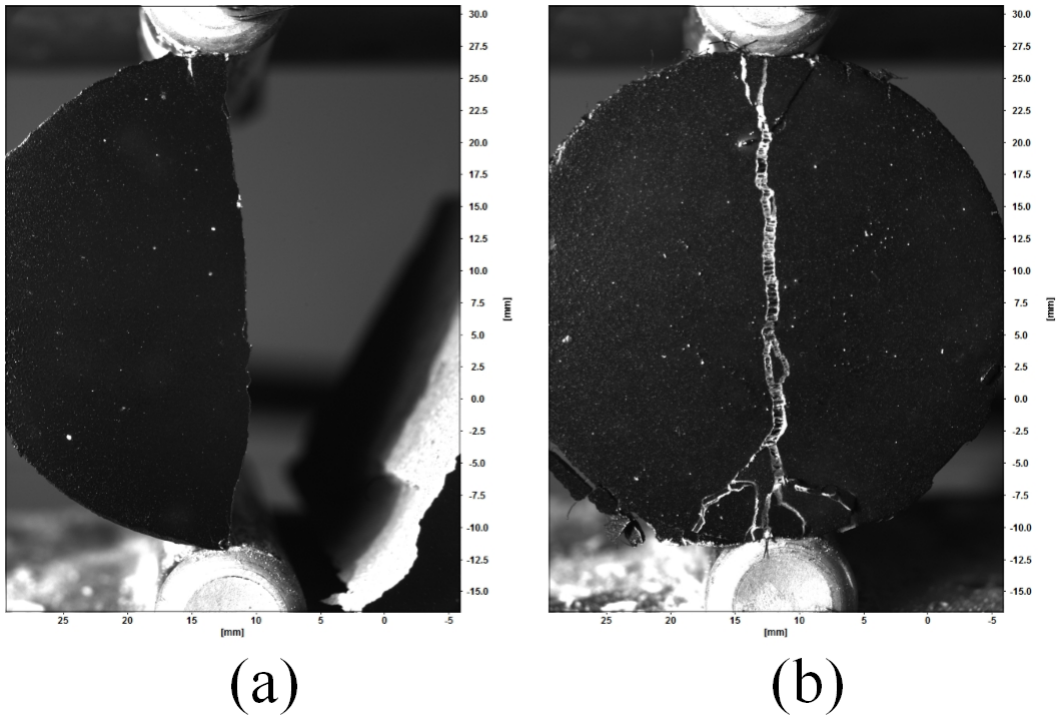
Cement sample failure (a) Oil Well G cement (b) Oil Well G cement with fiber reinforcement.

**Fig 9:**
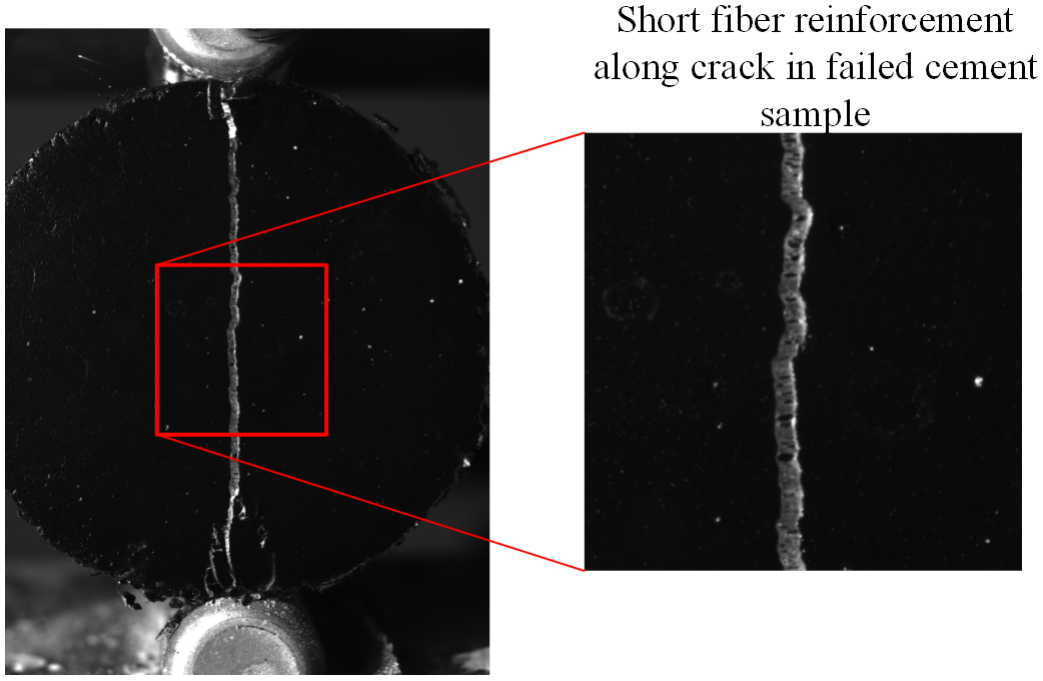
Example fiber reinforced cement sample with fibers preventing the complete failure of the cement sample.

The three short fiber reinforcements used in this study were tested in addition to cement samples without reinforcement. An example of a failed Oil Well G cement sample is shown in Fig 8(a). This figure shows the complete failure of the Oil Well G cement sample. For comparison purposes, a fiber reinforced cement sample is shown in Fig 8(b). A close up image (Fig 9) of a failed fiber reinforced cement samples shows the presence of fibers oriented across the cement sample crack.

**Fig 10:**
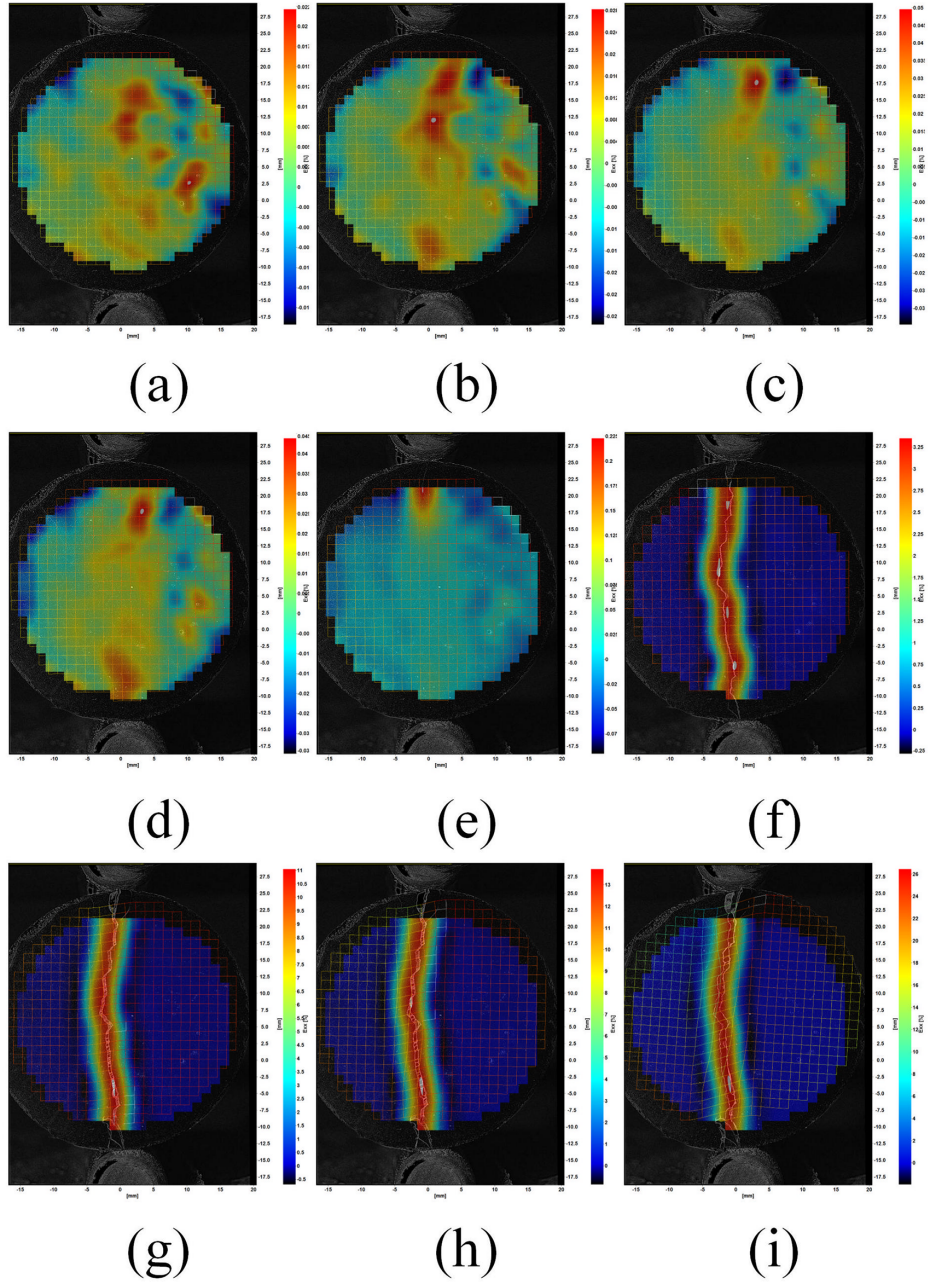
Representative strain field for a fiber reinforced cement sample (a) Maximum strain 0.022% (b) maximum strain 0.028% (c) maximum strain 0.05% (d) maximum strain 0.045% (e) maximum strain 0.225% (f) maximum strain 3.25% (g) maximum strain 11% (h) maximum strain 13% (i) maximum strain 26%.

Representative strain fields for a fiber reinforced cement sample are shown in Fig 10. This image sequence demonstrates the change in strain in the *x*-direction as a compressive load is applied to the cylindrical sample. Images (a) to (d) show the increase in strain, *ε*_*xx*,_ along the loading axis (*y*-axis) of the sample. Image (e) shows the initial crack and failure of the sample and images (f) to (i) demonstrate the progressive increase in strain after failure has occurred. After failure, large displacements and strain occurs, however, the fiber reinforced cement sample does not completely fail.

**Fig 11:**
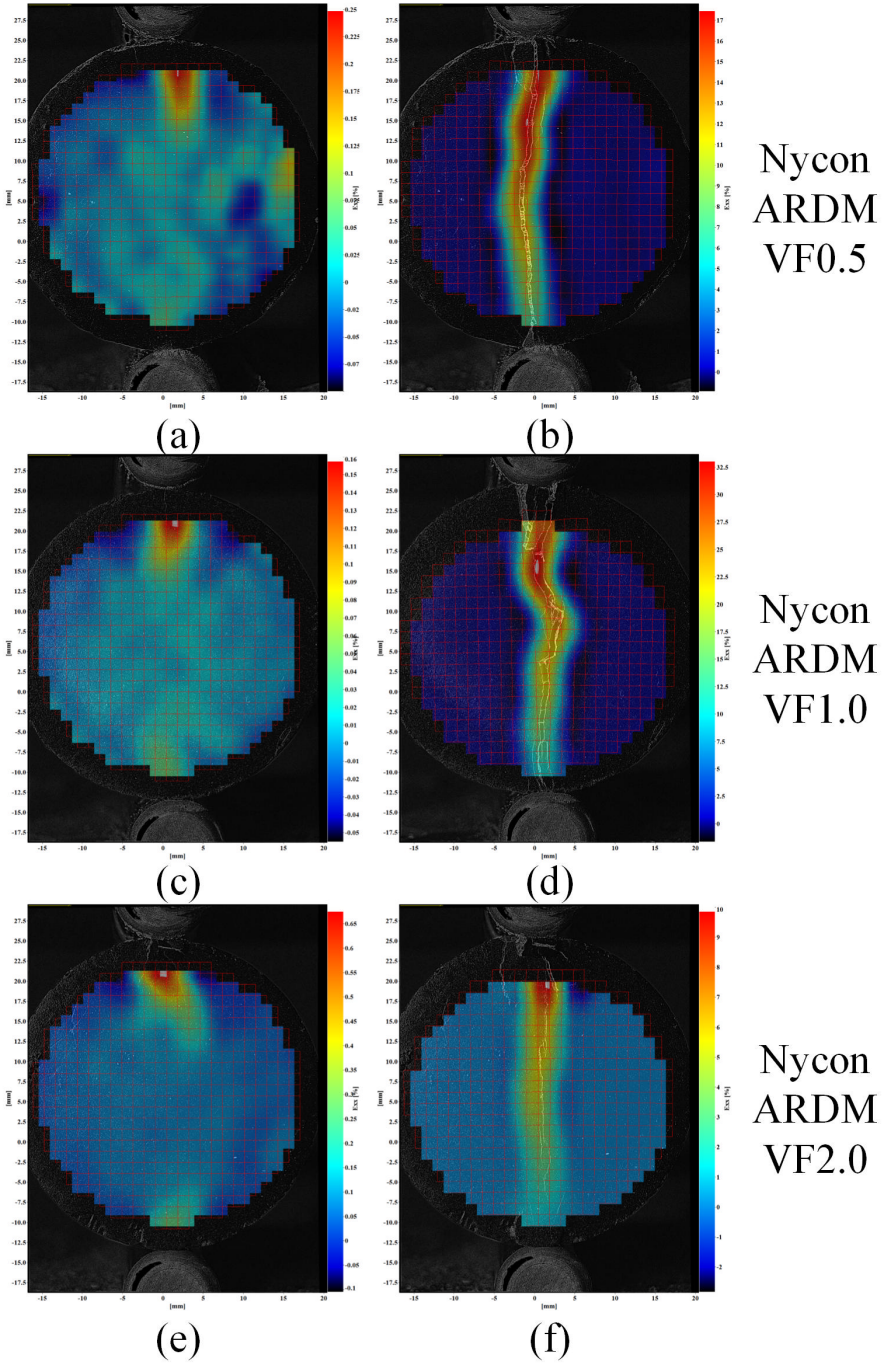
Example strain field of cement samples of Nycon ARDM fibers (left images strain prior to sample failure; right images maximum sample strain).

**Fig 12:**
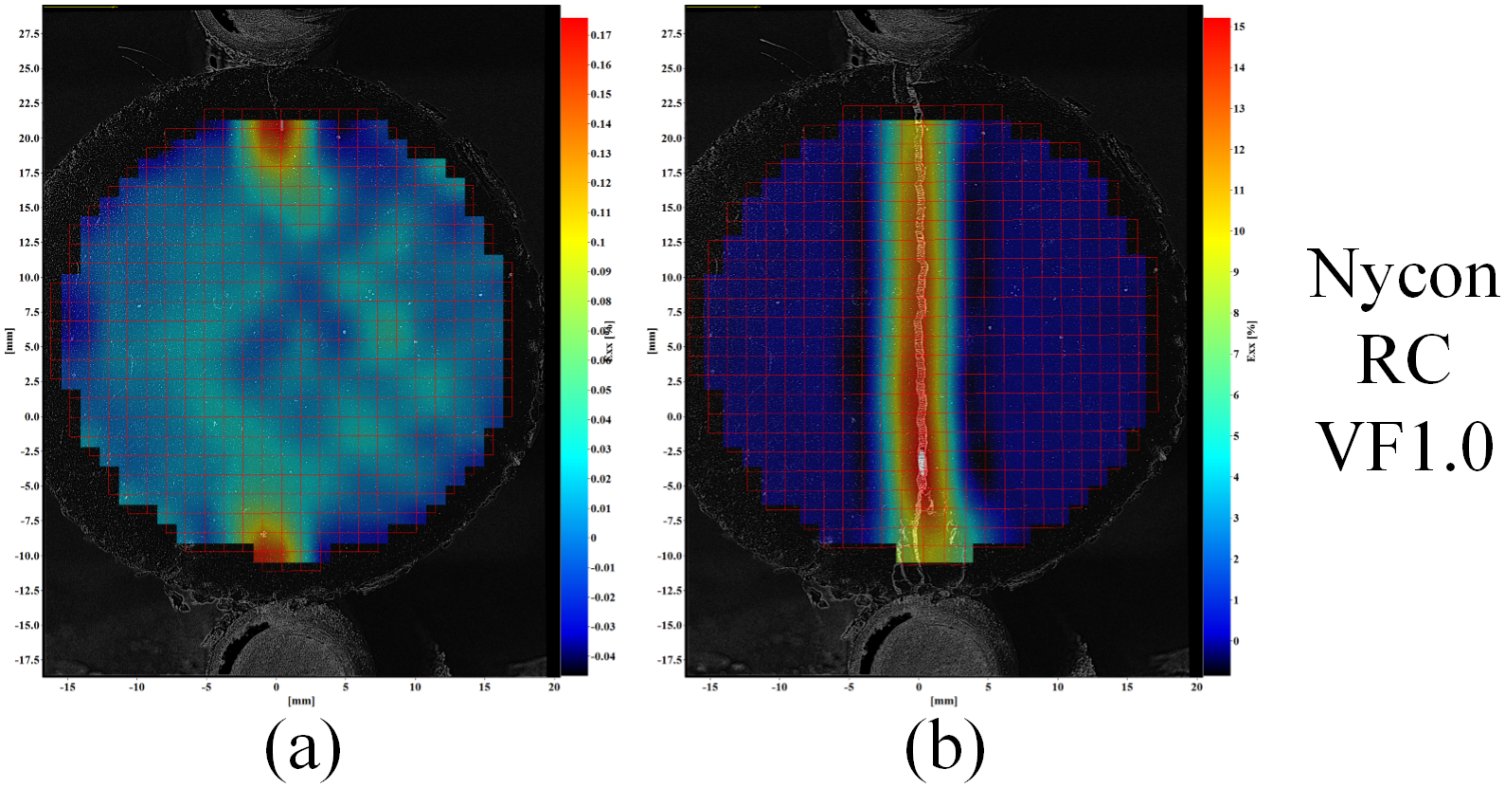
Example strain field of cement samples of Nycon RC Fibers. Left images strain prior to sample failure; right images maximum sample strain).

**Fig 13:**
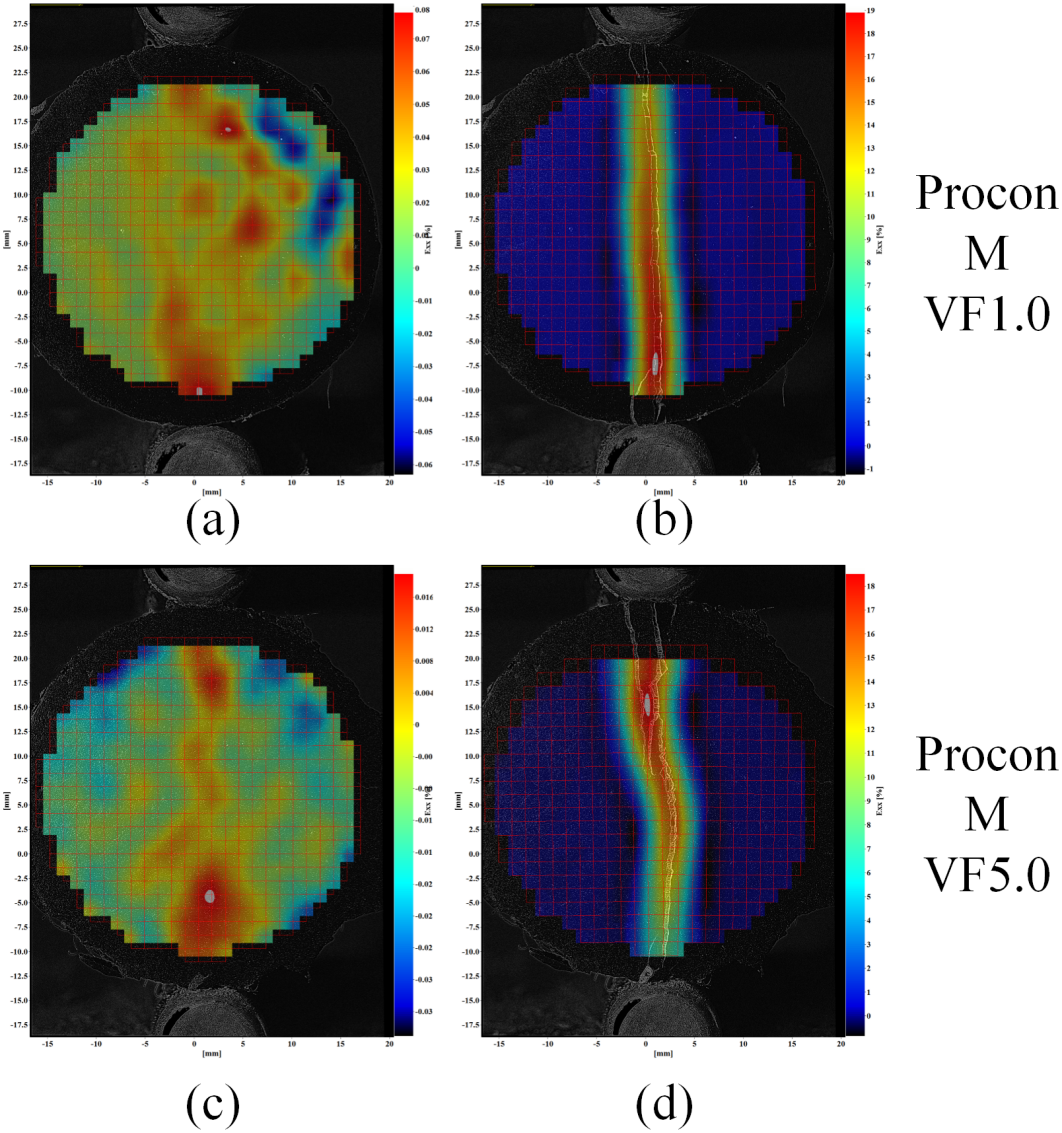
Example strain field of cement samples of Procon M Fibers. Left images strain prior to sample failure; right images maximum sample strain).

Example strain fields for *ε*_*xx*_ of the fiber reinforced cement samples are shown in Fig 11, Fig 12 and Fig 13 for the each fiber reinforcement and fiber volume fraction (VF) combinations. These figures show the sample strain prior to the cement sample cracking (left images) as well as the measured strain at the end of each sample test (right images).

**Fig 14:**
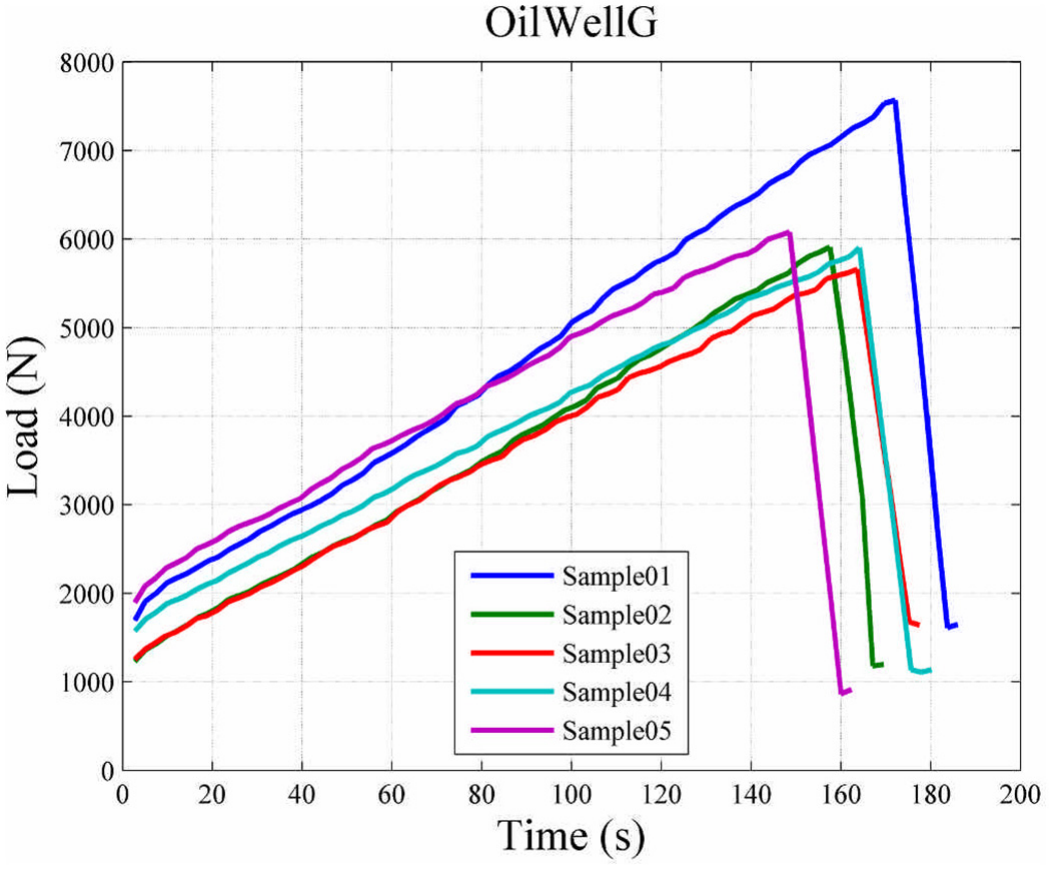
Load vs time curve for Oil Well G cement.

**Fig 15:**
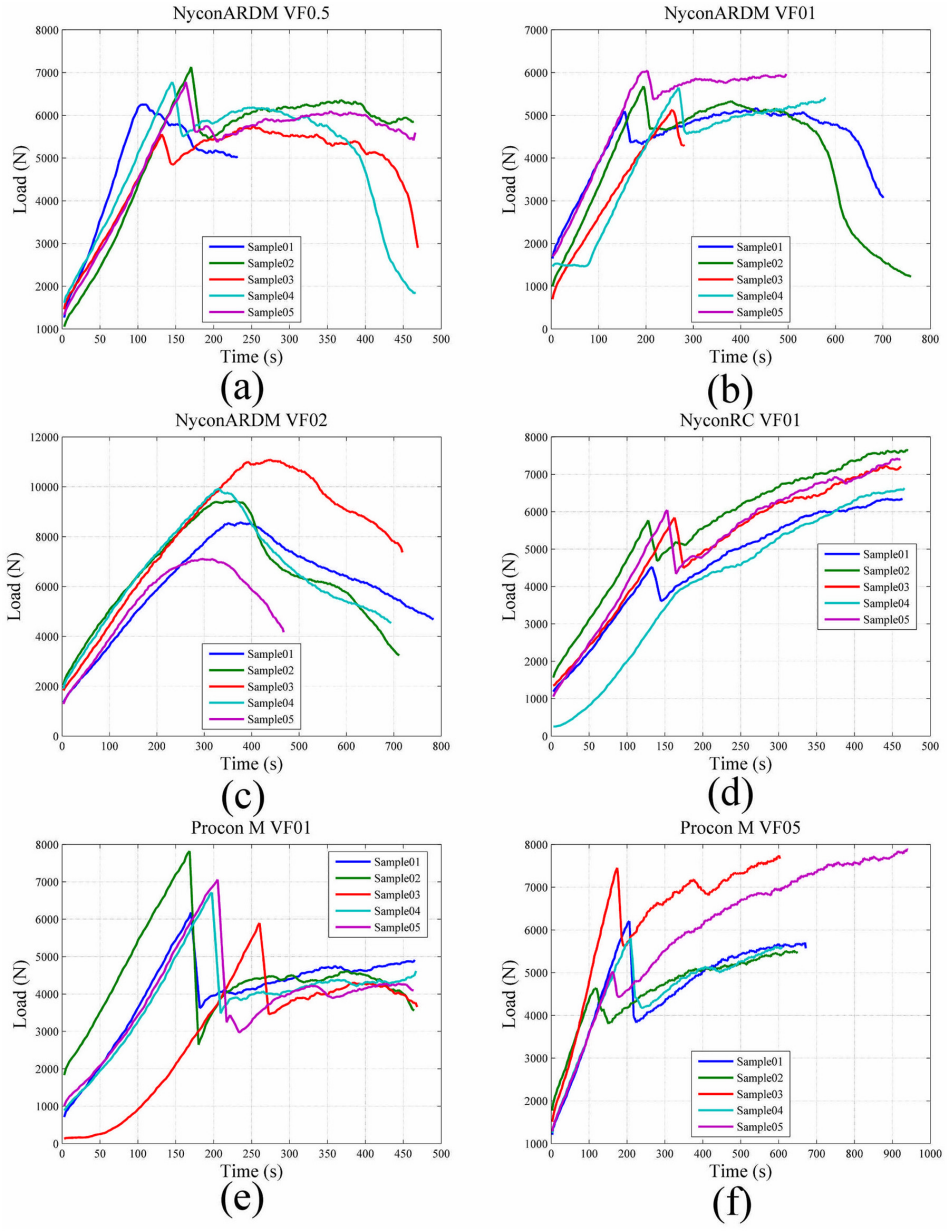
Comparison of load vs time curves for cement samples (a) Oil Well G samples, (b) Nycon ARDM VF0.5 samples (c) Nycon ARDM VF1.0 samples (d) Nycon ARDM VF2.0 samples (e) Nycon RC VF1.0 samples (f) Procon M VF1.0 samples (g) Procon M VF2.0 samples.

A load versus time curve for neat Oil Well G cement is shown in Fig 14. For comparison purposes, load versus time curves are shown in Fig 15 for the different fiber reinforcement and fiber volume fraction (VF) combinations used in this study.

**Fig 16:**
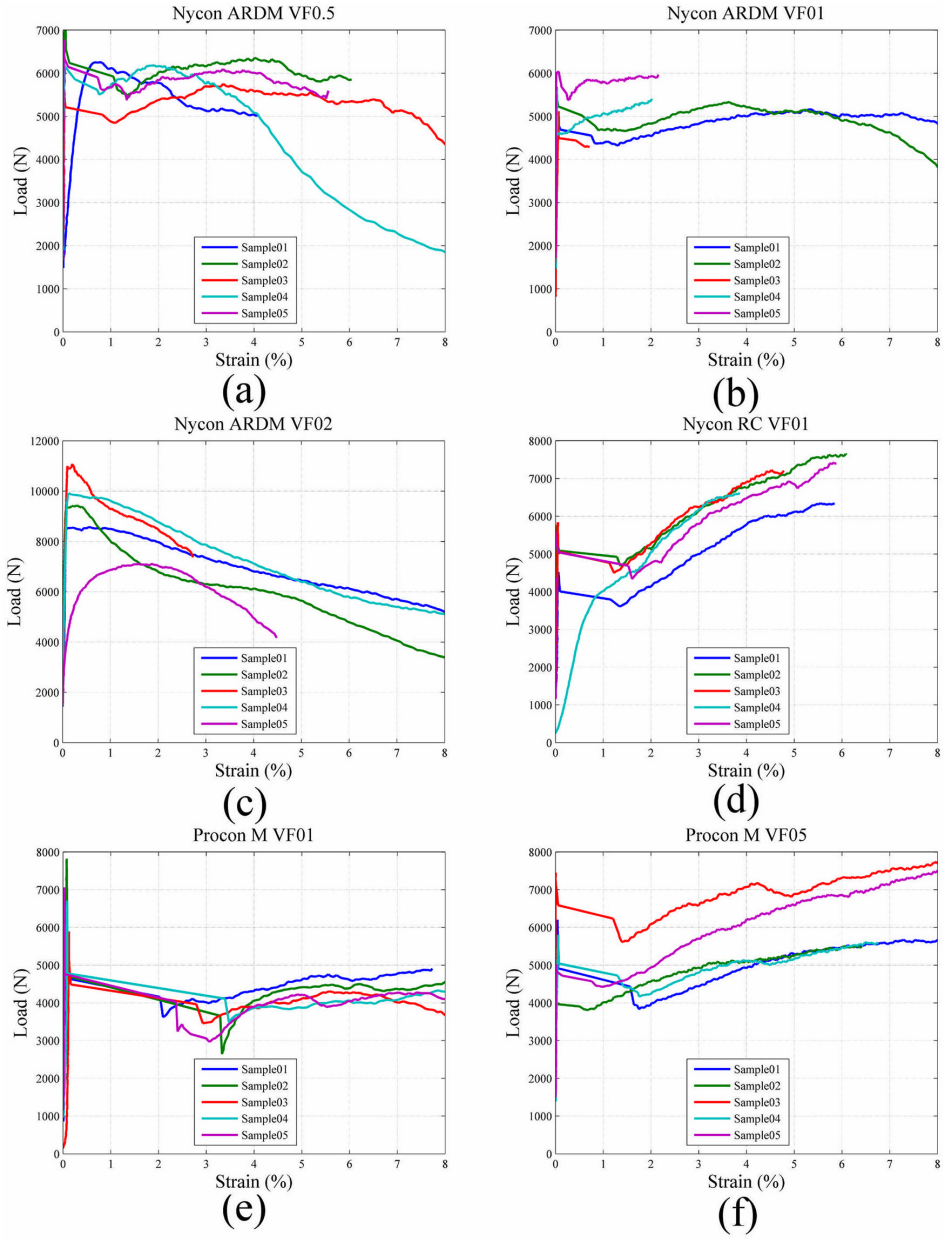
Comparison of load vs strain curves for cement samples (a) Nycon ARDM VF0.5 samples (b Nycon ARDM VF1.0 samples (c) Nycon ARDM VF2.0 samples (d) Nycon RC VF1.0 samples (e) Procon M VF1.0 samples (f) Procon M VF2.0 samples.

**Fig 17:**
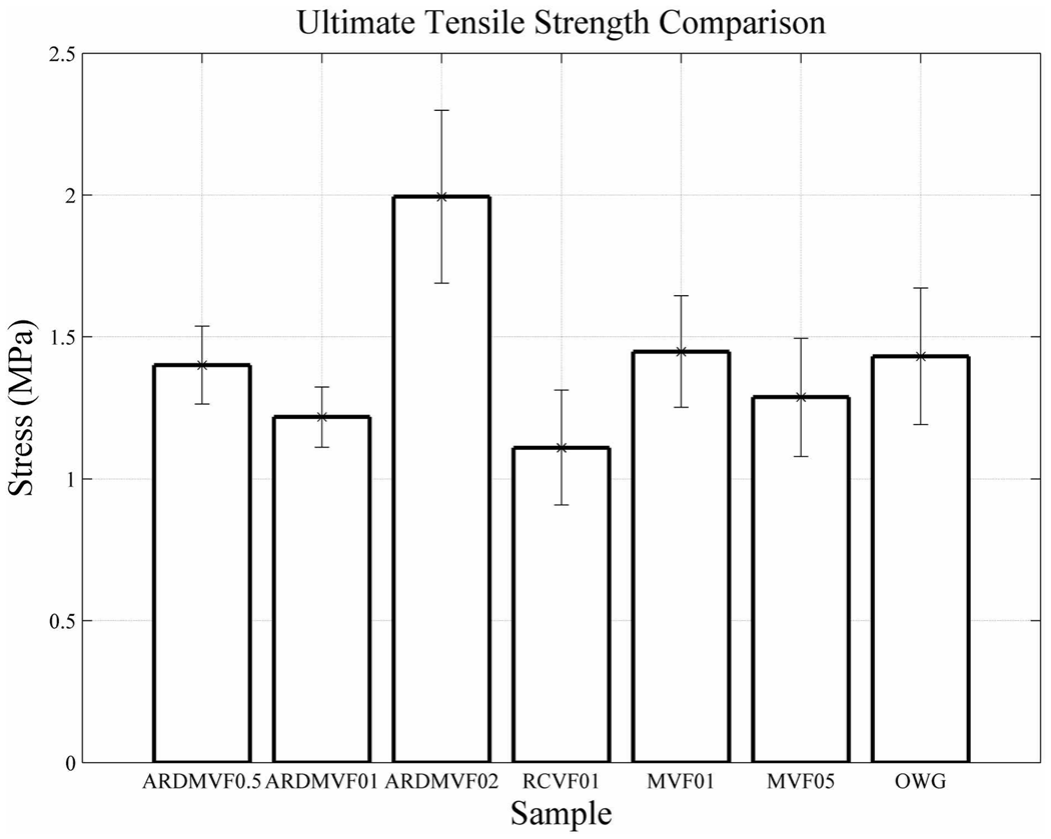
Fiber reinforced cement ultimate strength comparison.

For all test samples, the average tensile strain, *ε*_*xx*_, was plotted against the measured compressive load applied to the test sample. Comparison of the load versus strain curves for each fiber and volume fraction combination is shown Fig 16 and demonstrates the progressive failure behavior of each of the fiber reinforced cement samples. The figure demonstrates the continued load bearing capability of each after initial failure occurs. The ultimate strength for all cement samples was also determined using Eq 2. The comparison of cement sample average ultimate strength is shown in Fig 17.

The toughness of each fiber reinforced cement sample was estimated by calculating the area under the load vs strain curves shown in Fig 16. Toughness was calculated at 2% strain for all samples and compared in Table 3.

**Table 3:** Comparison of fiber reinforced cement toughness.

	Toughness (N mm/mm)					
Sample #	Nycon ARDM VF0.5	Nycon ARDM VF01	Nycon ARDM VF02	Nycon RC VF01	Procon M VF01	Procon M VF05
Sample1	11026.4	8819.7	16246.9	7636.9	150.2	8955.3
Sample2	11854.4	9619.0	15930.9	9904.5	441.5	8046.5
Sample3	9956.0	3056.5	18695.0	9669.2	331.8	12260.9
Sample4	11714.4	9963.7	17984.4	6890.9	244.2	9273.1
Sample5	11524.9	11453.9	12837.9	9471.7	84.2	9107.9
Average	11215.2	8582.6	16339.0	8714.6	250.4	9528.8
Standard Deviation	689.2	2892.0	2034.2	1215.5	127.1	1430.3

## Discussion

The purpose of this study was to study cement reinforcement using short fibers. Cements are brittle materials often tested using a splitting tensile test in order to determine ultimate tensile strength. Here, we further the testing capacity by using digital image correlation to observe the impact of adding short fibers to the cement matrix in terms of ductility and crack propagation and failure process.

The three short fiber reinforcements used in this study were tested in addition to cement samples without reinforcement. Each of the cement samples was evaluated using a splitting tensile-DIC measurement method. Several studies utilize a splitting tensile test to evaluate brittle materials like cement however this method is typically used to evaluate the splitting tensile strength of brittle materials [21, 22]. The DIC measurement method used in this study simplifies the sample instrumentation procedure and allows for strain to be measured across the entire sample surface.

Since a contract free measurement method has been used in this study, sample failure can be examined. Displacement-versus-time curves are shown in Fig 16 to demonstrate the effect of short fiber reinforcements. Fig 14(a) shows that the Oil Well G samples demonstrated a linear increase in stress until failure occurred. Similarly, the fiber reinforced cement samples (Fig 15(a)–15(f)) show a linear increase in load until failure. After failure there is a decrease in stress for each sample; however, there remains significant load-bearing capability after initial failure occurred. This indicates that the short fiber reinforcements prevent the complete failure of the cement samples. The behavior of the short fiber reinforced cement samples shown in Fig 15 demonstrates that the samples exhibit progressive failure. The progressive failure behavior occurs since the short fibers have an ultimate strength much greater than the cement matrix [23].

The strain fields in Fig 10 show that the splitting tensile-DIC test method can be used to evaluate the cylindrical test samples both prior to initial failure and post failure. Since sample strain can be measured after initial failure occurs, this method can be used to evaluate the toughness of different cement-short fiber combinations. The strain fields in Figs 11 to 13 show that similar strain behavior was exhibited for the three short fibers used in this study. These images show that prior to failure of the cement, strain is localized along the loading axis of the test sample. After failure occurs, the initial crack continues to expand throughout the sample as the compressive load is applied. High strain occurs along the length of the crack for these samples, however complete sample failure did not occur for any of the test samples. The high strains shown are due to the short fibers preventing complete sample failure.

Different quantities of short fibers and short fiber materials were used in this study. Despite varying quantities of fibers and fiber materials, the fiber reinforced cement samples exhibited similar behavior. One of the key similarities between fiber reinforced cement samples was their failure. The Oil Well G cement samples exhibited rapid and catastrophic failure when the ultimate strength of the cement was reached. In contrast, reinforcement of the fiber reinforced cement samples prevented catastrophic sample failure. All fiber reinforced cement samples exhibited similar behaviors, whereas there was initial failure of the sample and then progressive failure afterwards. Progressive failure of the fiber reinforced cement samples shows that the short fibers act as crack arrestors that prevent complete sample failure.

The short fiber reinforcements used in this study were compared by evaluating the toughness of each sample and by determining the ultimate tensile strength. Fig 17 shows that similar ultimate tensile strengths were found for all cement samples examined in this study. The addition of short fibers within a cement matrix should result in an increase in ultimate tensile strength when compared with neat cement [24]; however, the small sample size used in this study did not allow a significant increase in cement ultimate strength to be observed.

The toughness of each fiber reinforced sample was also evaluated (Table 3). The large variations in toughness could be attributed to improper fiber dispersion, but all result variability is consistent with typical findings in the literature for brittle material behavior. Fibers acted as good crack arrestors as expected, which is valuable in many applications where catastrophic failure would have disastrous repercussions. Using the indirect splitting tensile test replicates such worse case failure scenarios. Using the DIC system to track progression of the strains and failure provided valuable insight and new opportunities for future research in the area.

## Conclusions

Fiber reinforced cement samples were evaluated using an indirect splitting tensile method. This method was chosen since conventional mechanical testing methods are difficult to perform on brittle materials like cement. Conventional splitting tensile tests commonly measure strain using devices like strain gauges or extensometers however these devices only allow for strain to be measured at discrete locations. In addition, the deformation and strain of the cement sample was measured using a contact free 2D DIC optical measurement method. The full field DIC measurement method allows for strain to be measured across the entire surface of the cylindrical test sample. Additionally, splitting tensile tests are commonly used to determine the ultimate tensile strength of a material without measuring displacement or strain.

Three short fiber reinforcements were evaluated using the splitting tensile-DIC measurement method. The results from the DIC optical measurements demonstrate that the maximum strain occurs along the loading axis for all fiber reinforced cement samples. In addition, progressive failure was observed for all fiber reinforced cement samples. Since the DIC measurement method is a contact free measurement technique, this method can be used to measure the strains that occur at initial sample failure as well post failure strain.
